# Transfer learning and wavelength selection method in NIR spectroscopy to predict glucose and lactate concentrations in culture media using VIP‐Boruta

**DOI:** 10.1002/ansa.202000177

**Published:** 2021-04-05

**Authors:** Hiromasa Kaneko, Shunsuke Kono, Akihiro Nojima, Takuya Kambayashi

**Affiliations:** ^1^ Department of Applied Chemistry School of Science and Technology Meiji University Kawasaki Japan; ^2^ Research & Development Group Hitachi, Ltd. Yokohama Japan

**Keywords:** Boruta, culture medium, partial least‐squares, spectra analysis, transfer learning

## Abstract

Regression models are constructed to predict glucose and lactate concentrations from near‐infrared spectra in culture media. The partial least‐squares (PLS) regression technique is employed, and we investigate the improvement in the predictive ability of PLS models that can be achieved using wavelength selection and transfer learning. We combine Boruta, a nonlinear variable selection method based on random forests, with variable importance in projection (VIP) in PLS to produce the proposed variable selection method, VIP‐Boruta. Furthermore, focusing on the situation where both culture medium samples and pseudo‐culture medium samples can be used, we transfer pseudo media to culture media. Data analysis with an actual dataset of culture media and pseudo media confirms that VIP‐Boruta can effectively select appropriate wavelengths and improves the prediction ability of PLS models, and that transfer learning with pseudo media enhances the predictive ability. The proposed method could reduce the prediction errors by about 61% for glucose and about 16% for lactate, compared to the traditional PLS model.

## 1 INTRODUCTION

Process analytical technologies are important for nondestructive, real‐time, and quantitative analyses of various components in biopharmaceutical manufacturing processes. There are three types of vibrational spectroscopy: near‐infrared (NIR), mid‐infrared, and Raman spectroscopy. The focus of this study is NIR spectroscopy^1^ because influences of water absorption and fluoresces are relatively small and low‐cost monitoring tools. The use of NIR spectroscopy enables us to measure the major components, such as glucose and lactate, contained in culture media in‐line.^2^, ^3^, ^4^, ^5^, ^6^ Using data from culture media during the incubation process, a regression model can be constructed between the spectra matrix X and an objective variable y (eg, glucose and lactate concentrations), enabling the y‐values to be predicted from the X‐values in new culture media. Highly predictive models are required to control biopharmaceutical manufacturing processes using predicted y‐values. Although the predictive accuracy can be improved by increasing the variety of samples of culture media, the incubation period is more than one week, and the preparation of samples takes considerable time and effort. Therefore, a method of constructing regression models with high predictive accuracy from a small number of batches is required.

In this study, we use a batch culture process. In the culture media, X is measured with a Fourier transform NIR spectrometer, and y denotes the concentration of glucose or that of lactate. Regression models are then constructed between X and y. Linear regression analysis methods include partial least‐squares (PLS) regression,^7^ ridge regression, least absolute shrinkage and selection operator, and elastic nets,^8^ whereas nonlinear approaches include support vector regression,^9^ random forests (RFs),^10^ and deep neural networks.^11^ Because the relationship between spectra and concentrations basically follows a Beer–Lambert law,^12^ linear methods are often used to construct regression models. Although high correlations exist between wavelengths or wavenumbers in spectra, PLS allows the construction of a regression model with latent variables whose number is fewer than X variables,^6^ which is why it is often used in spectral analysis. Thus, PLS is the focus of this study.

In this paper, we discuss methods using culture medium samples (culture media) and wavelength selection methods to construct regression models with a small number of batches. To construct regression models with high predictive accuracy using only a small number of batches, the use of pseudo culture medium samples (pseudo media) has been considered.^13^, ^14^, ^15^, ^16^ In this study, we use pseudo media, which were mixtures of a fresh culture medium before cell culturing, the culture medium after culturing and glucose powder in various volumes, to support regression models, as both culture media and pseudo media are available. We use transfer learning (TL) ^17^ to improve the predictive ability of regression models by considering samples with different characteristics. In TL, knowledge and data of source domain data, which is related to target domain data, are transferred to improve predictive accuracy of models in the target domain. As a model‐based approach in TL, Shie et al combined TL with deep convolutional neural networks (CNNs) by conducting unsupervised CNN pre‐training and supervised fine‐tuning.^18^ Huyhg et al. extract tumor information from medical images via TL and CNNs.^19^ Wen et al. developed deep TL including a three‐layer sparse auto‐encoder for data‐driven fault diagnosis in industry.^20^ Wu et al. combined TL with a long‐short term memory recurrent neural network model for bearing fault diagnosis.^21^ However, regression analysis methods cannot be changed in model‐based approaches in TL. Since PLS is used in this study, an algorithm for transferring a dataset^22^ is focused, and it is shown that the transfer of pseudo media improves the predictive ability of regression models for the prediction of culture media.

Wavelength selection has been discussed as a means of improving the predictive performance of regression models. Examples include stepwise wavelength selection,^23^ stacked PLS (SPLS),^24^ moving window PLS,^25^, ^26^ and genetic algorithm‐based PLS.^27^ These methods basically select wavelengths to optimize some statistic such as *r* ^2^ after cross‐validation. Of course, the value of the statistic is improved after wavelength selection. However, the selected wavelengths will be overfitted to training data when cross‐validation is used to calculate the statistics, and the selected wavelengths will be overfitted to test or validation data when external validation is used to calculate the statistics. The predictive performance for completely new data is not considered in wavelength selection.

Boruta^28^ is a variable selection method based on RF importance that uses a pseudo X‐dataset generated by shuffling samples and selects important variables from the original X. As Boruta does not optimize any statistics, it is expected to reduce the occurrence of overfitting to training data in regression models. However, as RF is a nonlinear regression analysis method and RF importance is calculated based on nonlinear relationships between X and y, the X variables considered as important in nonlinear RF may differ from those that are important for linear regression analysis methods such as PLS.

Therefore, we propose a new Boruta algorithm to construct linear regression models with high predictive accuracy. While the original Boruta was based on variable importance in RF, the proposed method uses variable importance in projection (VIP) ^7^ in PLS. The proposed VIP‐Boruta method eliminates unnecessary wavenumbers and selects important wavenumbers, thereby improving the predictive ability of regression models. Although the number of samples in our dataset (see Section 3 for more details) is low, more robust will be constructed by using TL and VIP‐Boruta than the traditional models.

To verify the effectiveness of the proposed method, we analyze a dataset of both culture media and pseudo media, and confirm that the predictive ability of PLS models improves using TL and VIP‐Boruta.

## 2 METHODS

### 2.1 Transfer learning

**FIGURE 1:**
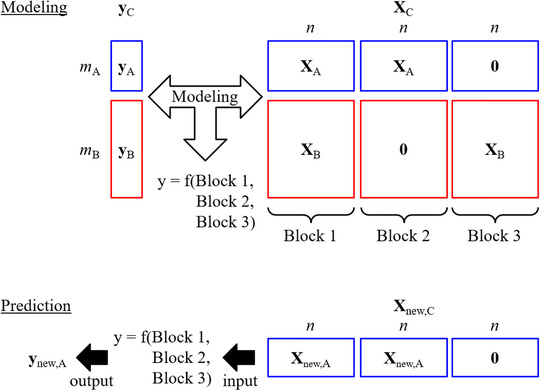
Basic concept of TL. A means culture medium, B means pseudo culture medium, and C means the combination of culture medium and pseudo culture medium in TL

Several TL algorithms exist. In this study, we focus on an algorithm for transferring a dataset.^22^ The new dataset **X**_C_ ∈ R^(^ *^m^* ^A+^ *^m^* ^B)×(3^ *^n^* ^)^ and **y**_C_ ∈ R^(^ *^m^* ^A+^ *^m^* ^B)×1^ is given as follows^22^:

(1) $$X_{C}=\left(\begin{array}{ccc} X_{A} & \;X_{A} & \;0\\ X_{B} & \;0 & \;X_{B} \end{array}\right)$$

(2) $$y_{C}=\left(\begin{array}{c} y_{A}\\ y_{B} \end{array}\right)$$

where **X**_A_ ∈ R*^m^* ^A×^ *^n^* is a spectra dataset of culture media, **y**_A_ ∈ R*^m^* ^A×1^ is a concentration dataset of culture media, **X**_B_ ∈ R*^m^* ^B×^ *^n^* is a spectra dataset of pseudo media, **y**_B_ ∈ R*^m^* ^B×1^ is a concentration dataset of pseudo media, *m*_A_ is the number of culture media, *m*_B_ is the number of pseudo‐culture media, and *n* is the number of wavenumbers. Figure 1 illustrates the basic concept of TL considered in this study. A regression model is constructed between the new dataset **X**_C_ and **y**_C_. The relationship between X and y that is common to both culture media and pseudo media will be trained in Block 1, that which is specific to culture media will be trained in Block 2, and that which is specific to pseudo media will be trained in Block 3. For example, when a linear regression model such as a PLS model^7^ is constructed between **X**_C_ and **y**_C_, the regression coefficient vector is split into three parts, the first part explaining the relationship between **X**_A_ and **X**_B_, and **y**_A_ and **y**_B_, the second part explaining the relationship in the culture media, and the third part explaining the relationship in the pseudo culture media.

In predicting new samples, Blocks 1 and 2 (spectra of culture media) and Block 3 (zero matrix) are combined (see Figure 1), which is given as follows:

(3) $$X_{\mathrm{new},C}=\left(\begin{array}{ccc} X_{\mathrm{new},A} & \;X_{\mathrm{new},A} & \;0 \end{array}\right).$$

Then, **X**_new,C_ is input into the regression model, allowing the y‐values to be predicted.

### 2.2 Boruta

Boruta^28^ is a variable selection method based on variable importance in RF.^10^ The samples of an original X‐dataset are shuffled, which means that the generated X‐variables are unrelated to y, before being added to the original dataset. The RF process is then conducted. The variable importance of the original and shuffled X are compared, and important variables are selected. The Boruta algorithm can be described as follows:

1. Copy the dataset of X.
2. Shuffle samples in the copied X‐dataset for each X‐variable. Because the new values are shuffled for each X‐variable, all the shuffled X‐variables are unrelated to y.
3. Combine the original X‐dataset with the shuffled X‐dataset, which means that both matrices are concatenated, and perform RF with y to calculate the importance of both the original X and the shuffled X.
4. Set the threshold value as the *p*‐percentile of variable importance in the shuffled X. In this study, *p* is set as 100.
5. Assign a “hit” for the original X‐variables whose importance exceeds the threshold value.
6. In repeating steps 3‐5, examine whether the original X‐variables are more important than the shuffled X‐variables with a two‐tailed test based on a binomial distribution.^29^ In this study, the significance level *α* is set as 0.05. The original X‐variables judged to be insignificant compared to the shuffled X‐variables in the iterations are removed.

We use the botura_py package^30^ in Python.

### 2.3 VIP‐Boruta

While the original Boruta^28^ uses variable importance calculated with RF,^10^ the proposed VIP‐Boruta instead uses the VIP^7^ in calculating PLS. The value of VIP for the *i*th X variable is given as follows:

(4) $$\mathrm{VI}P_{i}=\sqrt{\frac{n\sum_{j=1}^{a}\left\{\mathrm{SS}\left(q_{j}t_{j}\right)\left(w_{i,j}/\;{\left\|w_{j}\right\|}^{2}\right)\right\}}{\sum_{j=1}^{a}SS\left(q_{j}t_{j}\right)}}$$

(5) $$SS\left(q_{j}t_{j}\right)=q_{j}^{2}t_{j}^{T}t_{j}$$

where *n* is the number of X variables, **w** *_j_* is the weight vector of the *j*th LV, *w_i_*_,_ *_j_* is the value of the *i*th X variable for **w** *_j_*, *q_j_* is the y loading of the *j*th LV, **t** *_j_* is the score vector of the *j*th LV, and *a* is the number of LVs. The VIP‐Boruta algorithm can be described as follows:

1. Copy the dataset of X.
2. Shuffle samples in the copied X‐dataset for each X‐variable. Because the new values are shuffled for each X‐variable, all the shuffled X‐variables are unrelated to y.
3. Combine the original X‐dataset with the shuffled X‐dataset, which means that both matrices are concatenated, and perform PLS with y to calculate VIP of both the original X and the shuffled X. The number of LVs is optimized to maximize r^2^ after cross‐validation.
4. Set the threshold value as the *p*‐percentile of VIP in the shuffled X. In this study, *p* is set as 100.
5. Assign a “hit” for the original X‐variables whose VIP exceeds the threshold value.
6. In repeating steps 3‐5, examine whether the original X‐variables are more important than the shuffled X‐variables with a two‐tailed test based on a binomial distribution.^29^ In this study, the significance level *α* is set as 0.05. The original X‐variables judged to be insignificant compared to the shuffled X‐variables in the iterations are removed.

By using VIP to determine the variable importance, the X‐variables that are important for a PLS model are selected. The other parts of VIP‐Boruta are the same as the original Boruta algorithm.

Traditional variable selection methods basically select wavelengths to optimize some statistic such as r^2^ after cross‐validation. The selected wavelengths will be overfitted to training data when cross‐validation is used to calculate the statistics, and the selected wavelengths will be overfitted to test or validation data when external validation is used to calculate the statistics. The predictive performance for completely new data is not considered in wavelength selection. Since VIP‐Boruta does not optimize any statistics, it is expected to reduce the occurrence of overfitting to training data in regression models. Furthermore, the X‐variables that are important for a PLS model can be selected by using VIP to determine the variable importance in VIP‐Boruta.

## 3 EXPERIMENTS

### 3.1 Samples

Culture medium samples and pseudo‐culture medium samples were prepared to construct regression models for glucose and lactate.

The culture medium was Dulbecco's Modified Eagle Medium (SIGMA‐Aldrich), which does not include glucose or glutamine. Glucose (SIGMA‐Aldrich), glutamine (Thermo Fisher), and serum (35−015‐CV, Corning) were added to the culture medium with final concentrations of 5−7 g/L, 0.58 g/L, and 5 vol%, respectively, before inoculating the cells.

Chinese hamster ovary (CHO) cells (CRL‐12445, ATCC) were grown in the culture media in a commercial spinner flask with 150 ml working volume. The CHO cells grew over seven days. The cultivation conditions were as follows: incubation temperature, 37°C; CO_2_ concentration, 5%; and agitation rate, ∼150 rpm. The culture medium was sampled from the spinner flask once a day.

Pseudo media were prepared by blending three kinds of materials: a fresh culture medium with added glutamine and serum, the culture medium after inoculating the cells over seven days and removing the cells using a filter of 0.22 μm diameter, and glucose powder. The pseudo media included 102 samples prepared with two batch cultures. The concentrations of glucose and lactate in the pseudo media were 0.87‐8.0 g/L and 0.12‐3.1 g/L, respectively (details are given in the appendix).

### 3.2 Measurement

A Fourier transform NIR spectrometer (Matrix‐F, Bruker) was used for absorbance measurements of the samples. Transmission spectra were collected from an optical cell with 1‐mm optical path length. The spectra in the range of 4000‐12000 cm^–1^ were collected with a spectral resolution of 4 cm^–1^, with 128 scans performed at room temperature. A background spectrum was collected in air. Reference concentrations of glucose and lactate were measured with an enzymatic analyzer (BioPAT Trace, Sartorius).

## 4 RESULTS AND DISCUSSION

Glucose and lactate concentrations were predicted from the NIR spectra of the culture media. The culture media were divided into 22 training samples and 23 test samples. In addition, 102 pseudo‐culture media were considered. The 23 test samples of small but diverse test data are necessary to test regression models for actual situations in which the models are used. There exist 22 samples available other than the 23 test samples. This small number of training samples is one of the motivations for using pseudo‐culture media to improve the predictive accuracy of regression models.

**FIGURE 2:**
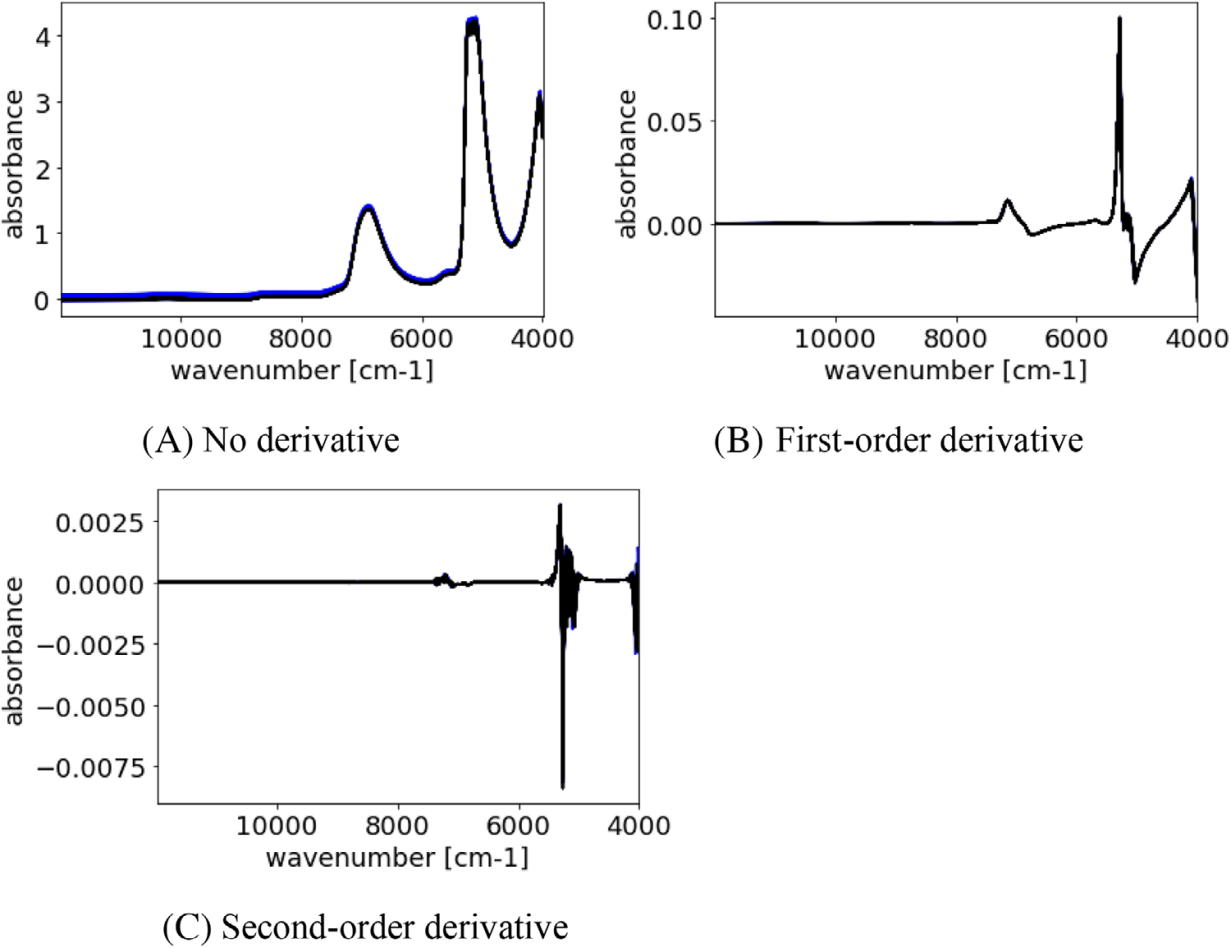
Preprocessed spectra of culture media and pseudo culture media. Blue and black lines mean spectra in culture media and spectra in pseudo culture media, respectively

The number of X‐variables in the spectra was 4148. The Savitzky–Golay (SG) method^31^ was used to preprocess the spectra. The window size was 21, the polynomial was of second order. Figure 2 shows preprocessed spectra of culture media and pseudo‐culture media. As shown Figure 2C, the second‐derivative spectra have large noise. In this study, first‐order derivatives were used. The number of LVs in PLS was optimized from 1 to 20 to maximize r^2^ after cross‐validation.

First, only the culture media were used to determine the wavelength selection. The following three methods were compared for the construction of regression models.

- PLS
- PLS + Boruta: PLS after wavelength selection with Boruta
- PLS + VIP‐Boruta: PLS after wavelength selection with VIP‐Boruta

**FIGURE 3:**
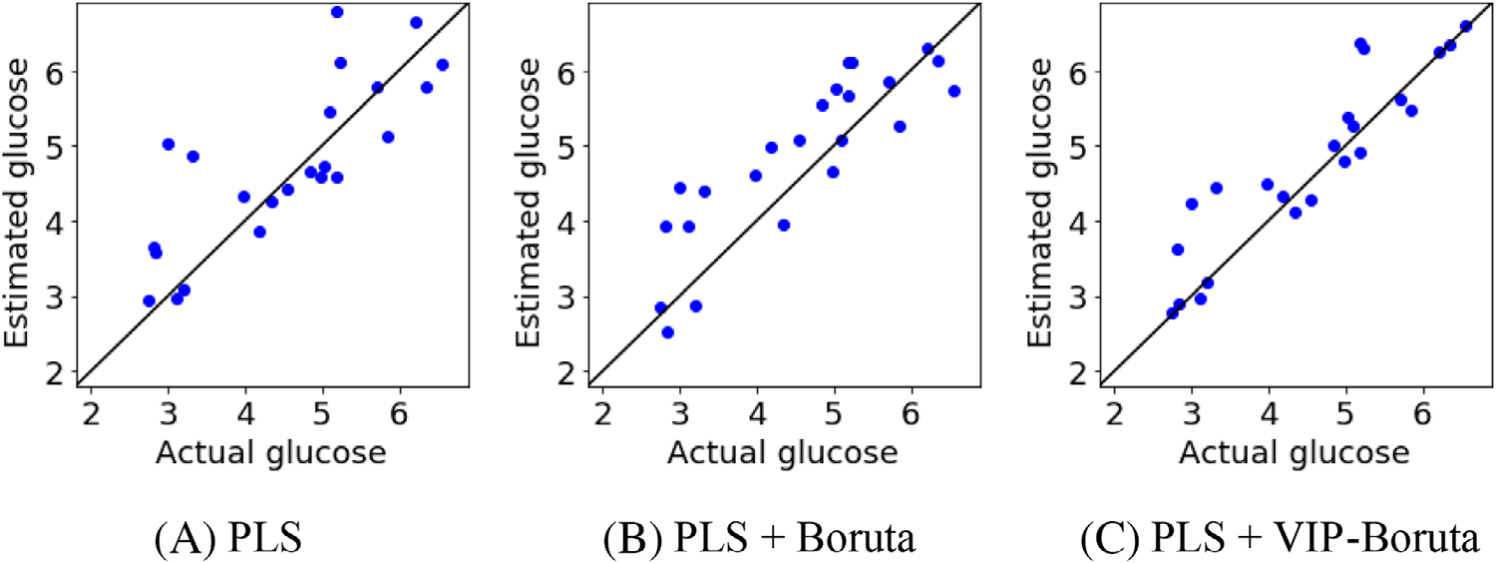
Measured and estimated values of glucose for test data when only culture media were used

**FIGURE 4:**
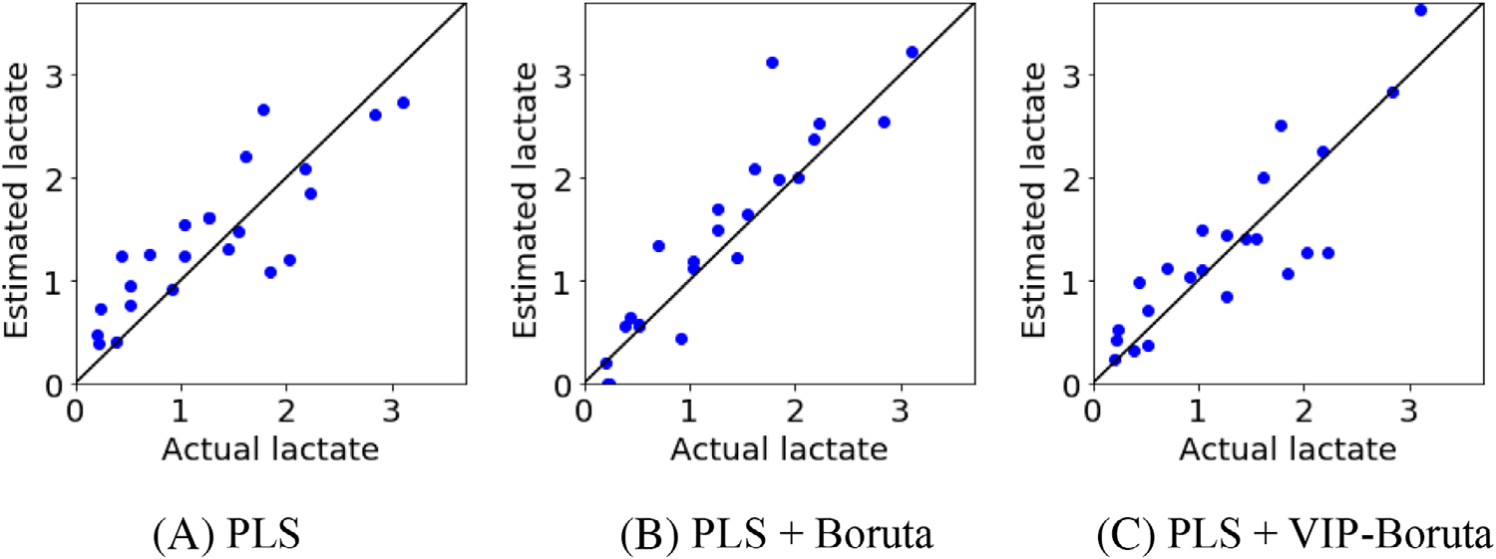
Measured and estimated values of lactate for test data when only culture media were used

Table 1 and Figure 3 show the predicted glucose concentrations in the test data for each method, and Table 2 and Figure 4 show those of the lactate concentrations. In comparing PLS + Boruta and PLS + VIP‐Boruta, both of which conduct wavelength selection before PLS analysis, the predictive ability of PLS + VIP‐Boruta is better for both glucose and lactate concentrations, achieving a higher *r* ^2^ and lower root mean square error (RMSE) than PLS + Boruta. Although r^2^ increased and RMSE decreased by using PLS + VIP‐Boruta, the improvement was not so clear From Figure 4 in lactate. As shown in Figure 3C, the samples are close to the diagonal lines, and glucose concentrations are accurately predicted by the proposed method. For the glucose concentrations, in particular, the proposed method predicts high and low glucose concentrations more accurately than the traditional PLS + Boruta method. This is because VIP‐Boruta, a linear variable selection method, is more appropriate for wavelength selection than Boruta, a nonlinear variable selection method, for linear regression analysis methods such as PLS.

**TABLE 1:** r^2^ and RMSE for test data in glucose when only culture media were used

	# of LVs	r^2^	RMSE
PLS	6	0.589	0.766
PLS + Boruta	1	0.667	0.690
PLS + VIP‐Boruta	5	0.792	0.545

**TABLE 2:** r^2^ and RMSE for test data in lactate when only culture media were used

	# of LVs	r^2^	RMSE
PLS	8	0.688	0.458
PLS + Boruta	12	0.777	0.387
PLS + VIP‐Boruta	15	0.815	0.352

Next, pseudo culture media were used in addition to culture media. The following four methods were compared for the construction of regression models.

- PLS
- PLS + TL: PLS in which pseudo‐culture media were transferred to culture media as shown in Figure 1
- PLS + TL + Boruta: PLS + TL after wavelength selection with Boruta for the dataset in Figure 1
- PLS + TL + VIP‐Boruta: PLS + TL after wavelength selection with VIP‐Boruta for the dataset in Figure 1

**FIGURE 5:**
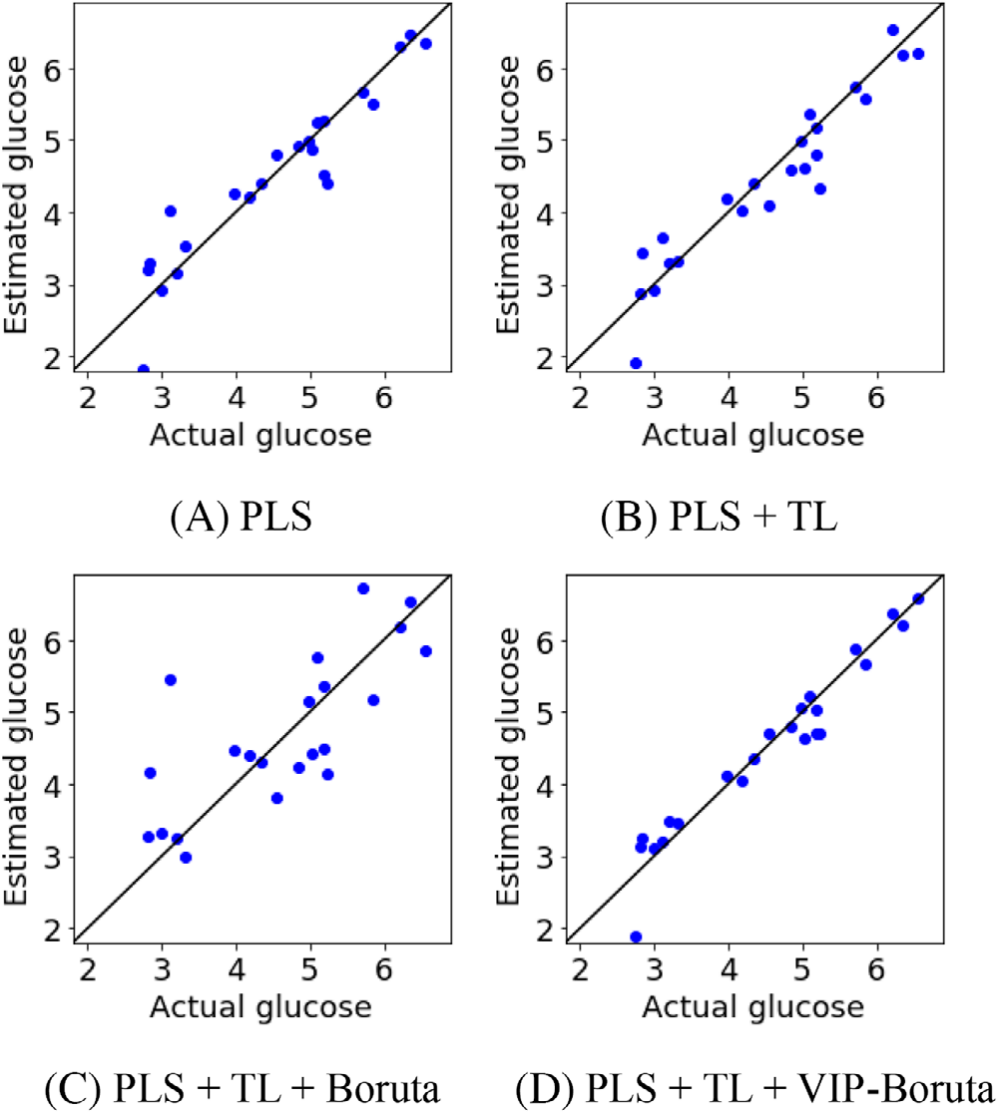
Measured and estimated values of glucose for test data when both culture media and pseudo media were used

**FIGURE 6:**
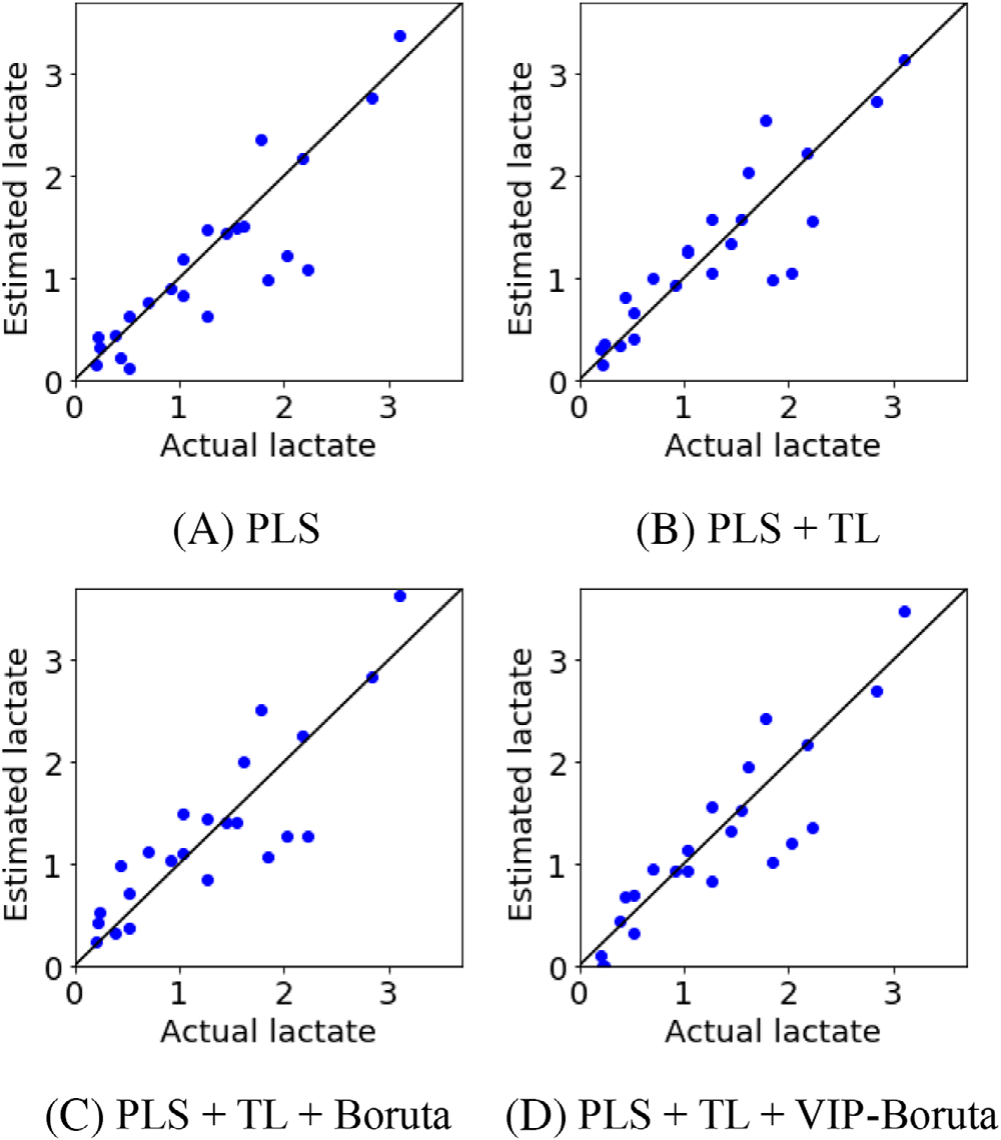
Measured and estimated values of lactate for test data when both culture media and pseudo culture media were used

Table 3 and Figure 5 show the predicted glucose concentrations in the test data for each method, and Table 4 and Figure 6 show those of the lactate concentrations. From Tables 1, 2, 3, 4, for both glucose and lactate concentrations, the high *r* ^2^ and low RMSE for PLS with both culture media and pseudo media, compared with the values for PLS with only culture media. Furthermore, PLS + TL produces higher r^2^ and lower RMSE values than PLS alone, confirming that PLS combined with TL improves the predictive performance for glucose and lactate concentrations although the improvement of RMSE was 6%.

**TABLE 3:** r^2^ and RMSE for test data in glucose when both culture media and pseudo media were used

	# of LVs	r^2^	RMSE
PLS	20	0.888	0.400
PLS + TL	20	0.900	0.377
PLS + TL + Boruta	19	0.510	0.837
PLS + TL + VIP‐Boruta	10	0.939	0.296

**TABLE 4:** r^2^ and RMSE for test data in lactate when both culture media and pseudo media were used

	# of LVs	r^2^	RMSE
PLS	20	0.748	0.411
PLS + TL	20	0.777	0.387
PLS + TL + Boruta	9	0.730	0.426
PLS + TL + VIP‐Boruta	15	0.781	0.383

From Tables 3 and 4, along with Figures 5 and 6, it can be seen that *r* ^2^ decreases and RMSE increases for both glucose and lactate concentrations after wavelength selection with Boruta and TL, indicating a decrease in predictive accuracy, whereas r^2^ increases and RMSE decreases after wavelength selection with the proposed VIP‐Boruta, indicating an increase in predictive accuracy. Even when combined with TL, VIP‐Boruta seems to be more appropriate for wavelength selection than Boruta for PLS analyses. Although r^2^ increased and RMSE decreased by using PLS + TL + VIP‐Boruta, the improvement was not so clear From Figure 6 in lactate. Figure 5D shows that the samples are close to the diagonal lines when using the proposed method, indicating accurate predictions of glucose concentrations. Thus, it has been confirmed that the proposed VIP‐Boruta can effectively select appropriate wavenumbers.

Comparing Tables 1 and 3, PLS + TL + VIP‐Boruta gives the highest r^2^ and the lowest RMSE for glucose concentrations, indicating the highest predictive accuracy. As shown in Figure 5D, while one sample is far from the diagonal, the others are close to the diagonal line, indicating that the glucose concentrations are predicted with high accuracy. A predictive PLS model could be constructed by transferring pseudo media and selecting the wavenumbers with VIP‐Boruta. However, comparing Tables 2 and 4, the r^2^ of PLS + TL + VIP‐Boruta is lower than that of PLS + VIP‐Boruta, and the RMSE of PLS + TL + VIP‐Boruta is higher than that of PLS + VIP‐Boruta. Therefore, the predictive ability of the PLS model is negatively affected by combining VIP‐Boruta with TL in lactate concentrations, although both PLS + TL + VIP‐Boruta and PLS + VIP‐Boruta are proposed methods. The cell culture media contain nutrients of amino acids and glucose, and metabolites such as lactate and ammonia. The concentrations of these components would correlate each other because the metabolites are produced while the nutrients are consumed during cell culturing. When there are high correlations among the components in the culture media, it could lead to unreliable and unstable prediction because the prediction model of the target material is affected by concentrations of other materials. Glucose was added to the pseudo‐culture media used in this research; therefore, it is considered that the correlation between the glucose and other components in the media would be decreased. By contrast, lactate was not added, and the correlation between the lactate and other components (other than glucose) in the media was not decreased. It causes the higher prediction accuracy for glucose concentration compared to the that of lactate.

In addition, the Boruta algorithm, in which the samples in X are shuffled, does not work effectively with TL as a result of the zero matrix shown in Figure 1 and RF in Boruta calculates variable importance considering nonlinear relationships between X and y whereas linear PLS models are constructed after wavelength selection. Additionally, TL simply triples the number of wavenumbers, making it easy for overfitting to occur. The wavenumbers selected for Boruta and VIP‐Boruta are discussed in the following paragraphs.

**FIGURE 7:**
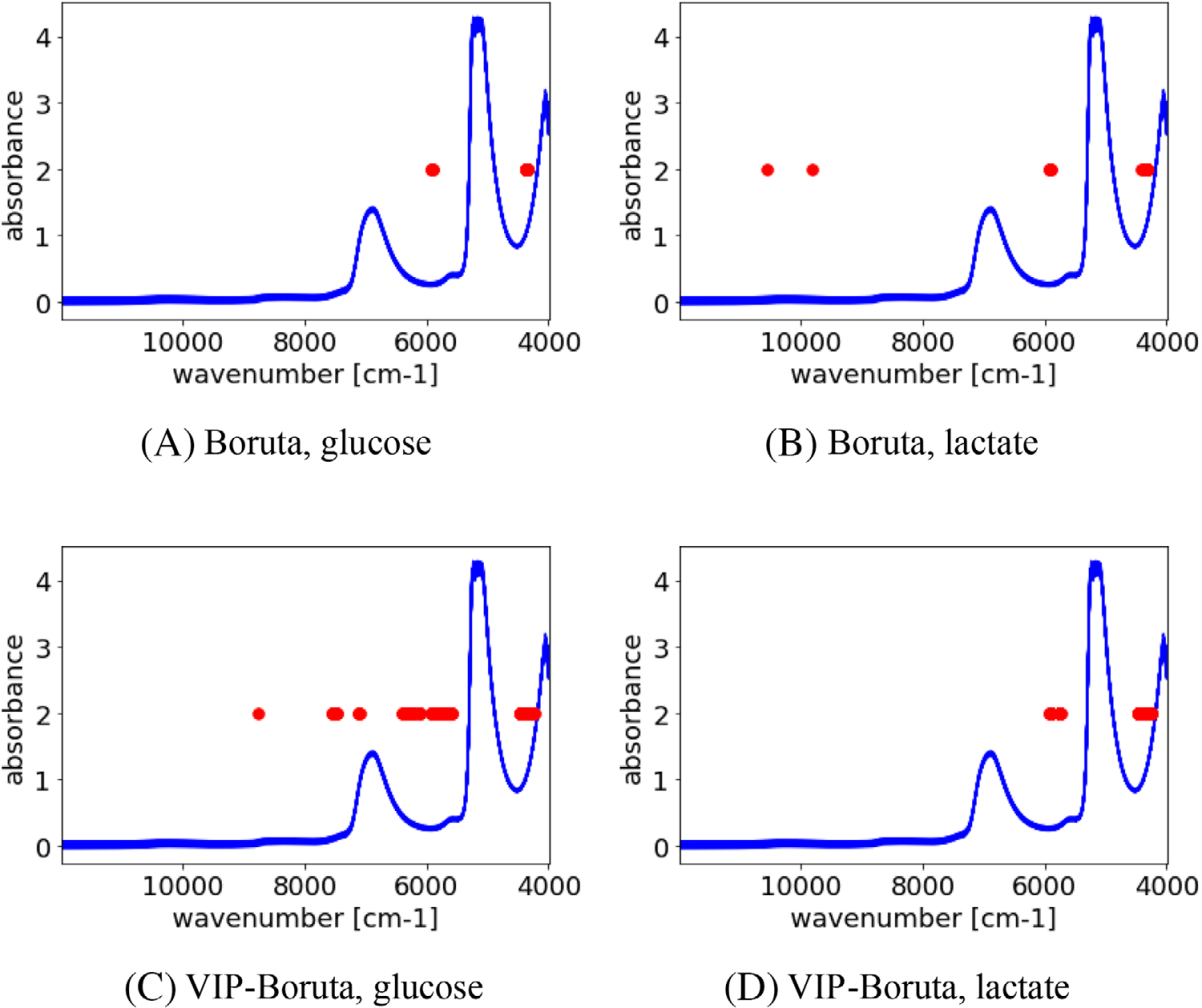
Selected wavenumbers in Boruta and VIP‐Boruta. Blue lines mean spectra in culture media, and red points denote selected wavenumbers

The wavenumbers selected with Boruta and VIP‐Boruta are shown in Figure 7. The vertical axis shows the absorbance, and the horizontal axis shows the wavenumber. The red circles represent the wavenumbers selected with the Boruta and VIP‐Boruta methods. The two peaks at around 7000 cm^–1^ and 5100 cm^–1^ are attributed to the absorption of water, which is the main component of the cell culture media. The clear absorption peaks due to the glucose and lactate bands cannot be observed as shown in Figure 7. These bands are weak and overlapped other peaks from other nutrition and metabolites in the culture medium. The absorption peaks expected from the functional groups of glucose and lactate^32^ and the wavenumbers selected with Boruta and VIP‐Boruta are listed in Tables 5 and 6, respectively. For glucose, several absorption peaks (8873–8547, 4400, 4386 cm^–1^) that were not selected with Boruta were selected with VIP‐Boruta. This result suggests that more peaks specific to glucose can be selected with VIP‐Boruta, indicating that the prediction ability can be improved with VIP‐Boruta. For lactate, several absorption peaks that are not specific to lactate (10561, 9816 cm^–1^) were selected with Boruta, but were not selected with VIP‐Boruta. In other words, VIP‐Boruta eliminated the wavenumbers irrelevant to the lactate concentration and improved the prediction ability. These results demonstrate that the VIP‐Boruta method can select relevant wavenumbers, eliminate wavenumbers irrelevant to the target values, and improve the prediction ability.

**TABLE 5:** Optical absorption bands associated with (a) glucose and (b) lactate molecular structures

(a) Glucose	
Spectra–Structure Correlation	Wavenumber (cm^–1^)
C‐H stretch third overtone	11834‐11390
O‐H second overtone (saccharides)	9811 and 10288
C‐H stretching second overtone	8873‐8547
C‐H stretching first overtone	5917‐5698
C = O and C‐O stretching	4762
O‐H bending & C‐O stretching combination	4762
O‐H stretching & C‐O stretching combination	4400
C‐H stretching & CH2 bending	4386
C‐H stretching & CH2 bending combination	4283, 42924307
C‐H bending	4252

(b) Lactate	
Spectra–Structure Correlation	Wavenumber (cm^–1^)
C‐H stretching third overtone	11834‐11390
Non‐bonded second overtone	10000
C‐H stretching second overtone	8873‐8547
O‐H stretch (first overtone) & C‐O‐H bending (first overtone) combination	8200
O‐H stretching & C‐O stretching first overtone	8070
O‐H stretching & O‐C‐O bending first overtone	7600
O‐H non‐bonded first overtone	6920
O‐H stretching & C‐H stretching	6500
C‐H stretching first overtone	5917‐5698
O‐H stretching & C = O stretching combination	5290
C‐H stretching & C = O stretching combination	4630, 4695

**TABLE 6:** Selected wavenumbers in Boruta and VIP‐Boruta

	Selected wavenumber (cm^–1^)
(a) Boruta, glucose	5914‐5906, 4399–4342
(b) Boruta, lactate	10561, 9816, 5914–5901, 4414–4314
(c) VIP‐Boruta, glucose	8760, 7541–7514, 7468–7084, 6390–5594, 4466–4237
(d) VIP‐Boruta, lactate	5914‐5741, 4470–4239

**FIGURE 8:**
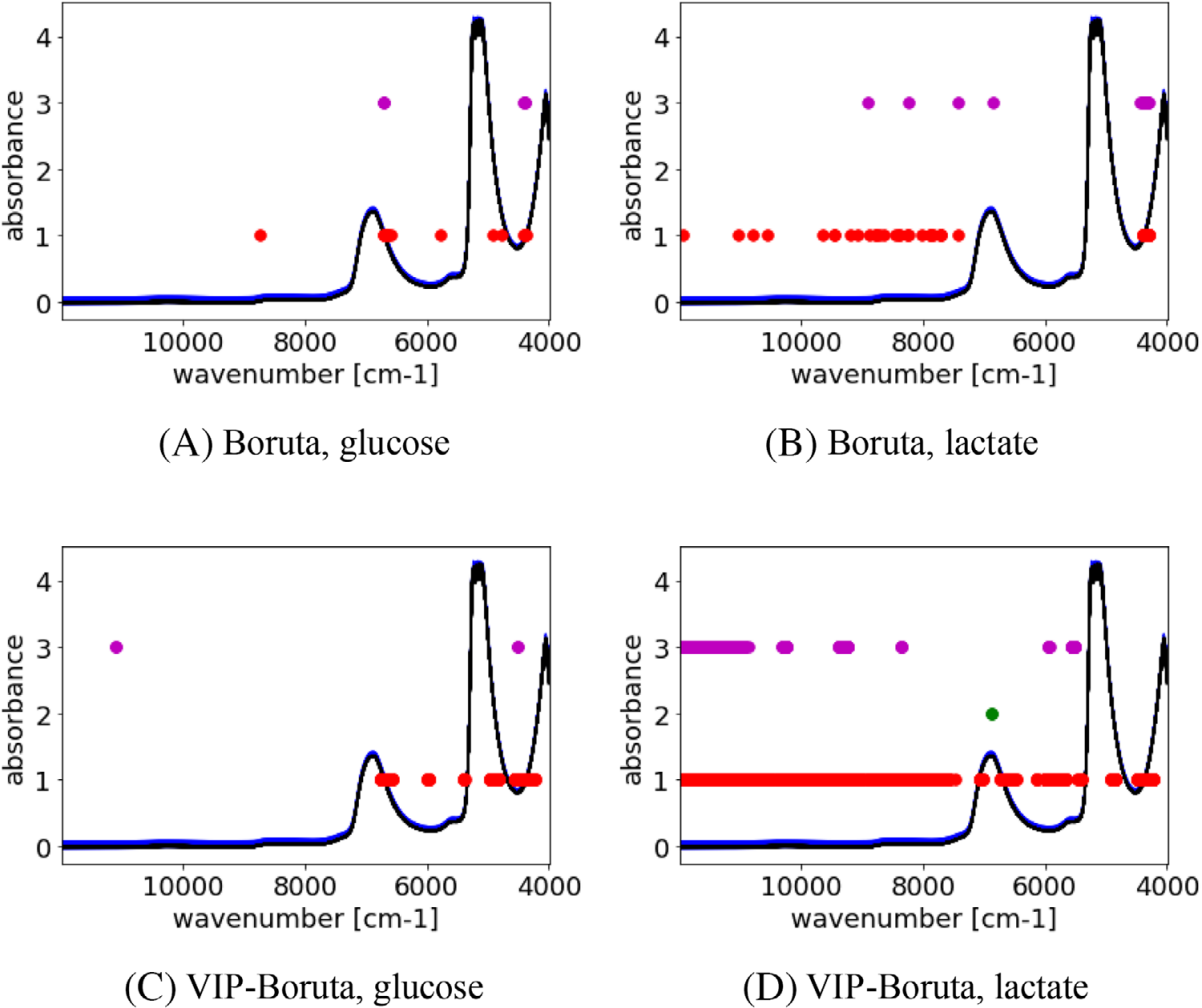
Selected wavenumbers in Boruta and VIP‐Boruta in TL. Blue and black lines mean spectra in culture media and spectra in pseudo culture media, respectively, and red, green, and magenta points denote selected wavenumbers of Blocks 1, 2, and 3, respectively (see Figure 1)

The wavenumbers selected with PLS + TL + Boruta and PLS + TL + VIP‐Boruta are shown in Figure 8 and Table 7. The vertical axis shows the absorbance, and the horizontal axis shows the wavenumber. The red, green, and magenta circles represent the selected wavenumbers in Blocks 1, 2, and 3, respectively. As in Figure 7, the two peaks at around 7000 cm^–1^ and 5100 cm^–1^ are attributed to water. TL selects additional wavenumbers compared with PLS + Boruta and PLS + VIP‐Boruta (see Table 5). About the few selected variables from Block 2 in Figure 8D, the peaks at around 7000 cm^–1^ is attributed to water. The additional wavenumbers selected with TL include low wavenumbers specific to glucose and lactate. Therefore, the application of TL improves the prediction ability.

**TABLE 7:** Selected wavenumbers in Boruta and VIP‐Boruta with TL

		Selected wavenumber (cm^–1^)
(a) Boruta, glucose	Block 1	8741, 6705–6580, 5774, 4914–4763, 4418–4379
	Block 3	6705‐6703, 4418–4381
(b) Boruta, lactate	Block 1	11950, 11034–10546, 9654–7438, 4403–4296
	Block 3	8919, 8228, 7438, 6857, 4441–4302
(c) VIP‐Boruta, glucose	Block 1	6761‐6572, 5990–5970, 5407–5384, 4968–4821, 4568–4234
	Block 3	11109‐11108, 4518–4516
(d) VIP‐Boruta, lactate	Block 1	11960‐7484, 7079–7027, 6728–6470, 6140–5405, 4915–4840, 4491–4223
	Block 2	6886‐6884
	Block 3	11960‐10876, 10309–10238, 9384–9239, 8361–8356, 5941–5934, 5565–5513

Comparing Figures 7D and 8D, the number of wavelengths selected with PLS + TL + VIP‐Boruta is much higher than with PLS + VIP‐Boruta in lactate. This would be because the small variance of lactate and high correlation with other components (as discussed in a previous paragraph), compared to that of glucose, made it difficult to construct a regression model. In TL, the dataset becomes as shown in Figure 1, where the number of wavenumbers is tripled. In this situation, many wavenumbers persist after wavenumber selection with VIP‐Boruta, and the noise in these wavenumbers has a negative effect on the PLS models. This is more likely for PLS + TL + VIP‐Boruta than for PLS + VIP‐Boruta without TL, resulting in higher predictive ability for PLS + VIP‐Boruta than for PLS + TL + VIP‐Boruta in the case of lactate concentrations. Thus, the number of selected wavenumbers should be carefully checked.

## 5 CONCLUSIONS

This study examined the use of wavelength selection and TL to improve the ability of PLS models to predict glucose and lactate concentrations from NIR spectra in culture media. For wavelength selection, we developed VIP‐Boruta, a combination of Boruta and VIP in PLS, and for TL, we constructed PLS models by transferring pseudo media. The actual datasets of culture media and pseudo media were analyzed using the proposed methods, and it was confirmed that VIP‐Boruta and TL could enhance the predictive ability of PLS models for both glucose and lactate concentrations compared with traditional methods. Additionally, the selected wavenumbers were confirmed to be reasonable for the prediction of glucose and lactate concentrations. We confirmed that the proposed VIP‐Boruta wavelength selection method could effectively select wavenumbers and improve the predictive ability of PLS models, and that TL with pseudo media could also improve the predictive ability, for both glucose and lactate concentrations. The combination of TL and VIP‐Boruta achieved further improvement in the predictive accuracy of glucose concentrations, but the predictions of lactate concentrations were worse than when using VIP‐Boruta alone. This is because Boruta shuffles the X samples, and the zero matrix generated with TL (see Figure 1) is incompatible with this shuffling process. As a result, TL produces three times the original number of wavenumbers, making overfitting more likely to occur in variable selection. Variable selection with TL is a challenge for the future.

The proposed method can be used to construct models that accurately predict glucose and lactate concentrations even when the number of NIR spectral samples in training data is low. The proposed models are expected to promote process analytical technology and achieve highly accurate control in biopharmaceutical manufacturing processes.

## CONFLICT OF INTEREST

The authors have declared no conflict of interest.

## Supporting information

Figure S1 Reference values of glucose and lactate in pseudo‐samples in 1st and 2nd attempts

## Data Availability

The data that support the findings of this study are available from the corresponding author upon reasonable request.
