# Supplementary material for: Transfer learning and wavelength selection method in NIR spectroscopy to predict glucose and lactate concentrations in culture media using VIP‐Boruta
Source: Anal Sci Adv. 2021 Apr 5;2(9-10):470–9. doi: 10.1002/ansa.202000177 (PMC10989590; doi:10.1002/ansa.202000177)
Supplement: Supplementary file 1 — Figure S1 Reference values of glucose and lactate in pseudo‐samples in 1st and 2nd attempts [file ANSA-2-470-s001.docx]

Appendix


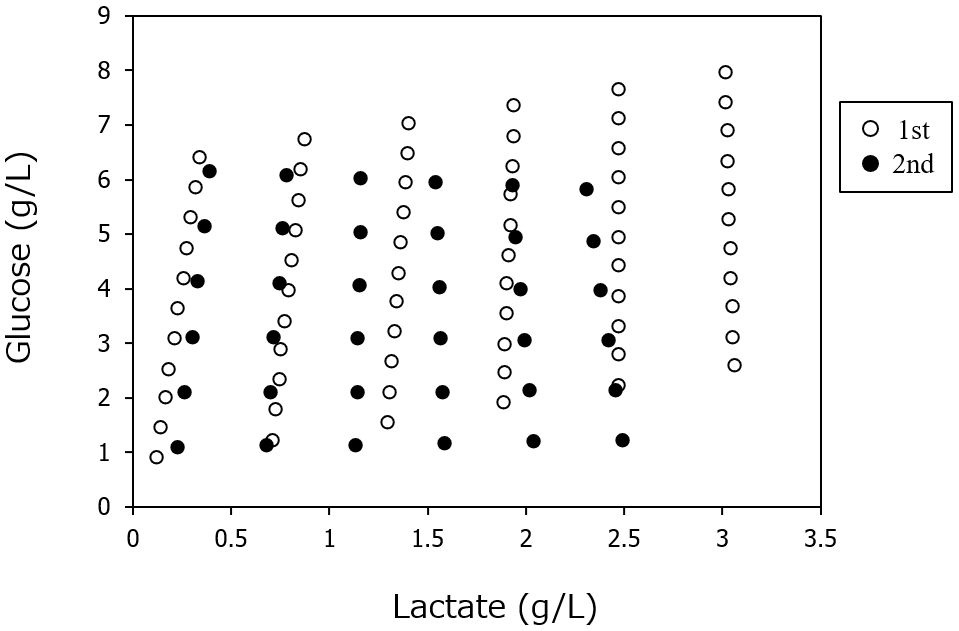


Figure S1. Reference values of glucose and lactate in pseudo-samples in 1st and 2nd attempts
